# Predictors of help-seeking behaviour in people with mental health problems: a 3-year prospective community study

**DOI:** 10.1186/s12888-021-03435-4

**Published:** 2021-09-03

**Authors:** Carolin M. Doll, Chantal Michel, Marlene Rosen, Naweed Osman, Benno G. Schimmelmann, Frauke Schultze-Lutter

**Affiliations:** 1 Department of Psychiatry and Psychotherapy, Medical Faculty, Heinrich-Heine-University and LVR clinic Düsseldorf, Bergische Landstr. 2, 40629 Düsseldorf, Germany; 2 Department of Psychiatry and Psychotherapy, Faculty of Medicine and University Hospital Cologne, University of Cologne, Cologne, Germany; 3 University Hospital of Child and Adolescent Psychiatry and Psychotherapy, University of Bern, Bern, Switzerland; 4 University Hospital of Child and Adolescent Psychiatry, University Hospital Hamburg-Eppendorf, Hamburg, Germany; 5 Department of Psychology and Mental Health, Faculty of Psychology, Airlangga University, Surabaya, Indonesia

**Keywords:** Help-seeking, Mental health problems, Longitudinal, Stigma, Structural equation model

## Abstract

**Background:**

The majority of people with mental illness do not seek help at all or only with significant delay. To reduce help-seeking barriers for people with mental illness, it is therefore important to understand factors predicting help-seeking. Thus, we prospectively examined potential predictors of help-seeking behaviour among people with mental health problems (*N* = 307) over 3 years.

**Methods:**

Of the participants of a 3-year follow-up of a larger community study (response rate: 66.4%), data of 307 (56.6%) persons with any mental health problems (age-at-baseline: 16–40 years) entered a structural equation model of the influence of help-seeking, stigma, help-seeking attitudes, functional impairments, age and sex at baseline on subsequent help-seeking for mental health problems.

**Results:**

Functional impairment at baseline was the strongest predictor of follow-up help-seeking in the model. Help-seeking at baseline was the second-strongest predictor of subsequent help-seeking, which was less likely when help-seeking for mental health problems was assumed to be embarrassing. Personal and perceived stigma, and help-seeking intentions had no direct effect on help-seeking.

**Conclusions:**

With only 22.5% of persons with mental health problems seeking any help for these, there was a clear treatment gap. Functional deficits were the strongest mediator of help-seeking, indicating that help is only sought when mental health problems have become more severe. Earlier help-seeking seemed to be mostly impeded by anticipated stigma towards help-seeking for mental health problems. Thus, factors or beliefs conveying such anticipated stigma should be studied longitudinally in more detail to be able to establish low-threshold services in future.

**Supplementary Information:**

The online version contains supplementary material available at 10.1186/s12888-021-03435-4.

## Background

Worldwide, mental disorders are an immense economic burden for society [1]. On average, 29.2% of adults will develop a mental illness in their lifetime [2]. The majority of people with a mental disorder do not seek help from any health care professional [3], although help-seeking for mental health problems (HSmental) at an early stage is crucial to reduce the burden of mental illness, and social and personal financial costs, to prevent future relapses, and to improve social functioning, and quality of life [4].

### *Help-seeking* for mental health problems and its predictors

HSmental is defined as an adaptive coping process that attempts to obtain external assistance to deal with mental health problems [5], including not only formal (e.g. psychiatrists) but also informal sources of help (e.g. friends) [5]. HSmental is predicted by different sociodemographic factors, such as older age [6, 7], female sex [6, 8], and lack of a current partner [9]. In addition, former positive help-seeking experiences [10], and more severe functional impairments [9], were positively correlated with HSmental. Furthermore, different types of stigma were identified as important barriers to HSmental [3, 10–13]. Thereby, stigma is commonly divided into structural stigma, perceived stigma, self-stigma, personal stigma, and anticipated stigma. While structural stigma is defined on a macro-social level as institutional policies and practices, societal-level conditions, cultural norms, and institutional practices that constrain the opportunities, resources, and well-being for stigmatized populations [14]; perceived stigma that might be perceived as part of structural stigma [14] is expressed on the micro-social level by the community’s prejudices and negative stereotypes towards people with a mental illness [15, 16]. Self-stigma is described as the affected persons’ internalisation of these stereotypes and prejudices [17], that were learned and hold in terms of personal stigma before developing a mental illness and identifying with the stigmatized group themselves [18]. Similar to this, personal stigma describes the unaffected individual’s own prejudice and negative stereotypes and it is often measured as the wish for social distance (WSD), which is basically the wish to avoid a specific group such as persons with a mental illness [19]. Furthermore, anticipated or perceived stigma [20] does not describe the experienced, but the anticipated stigmatization and discrimination by others in case one would become mentally ill oneself. This kind of stigma includes expectations that, for example, it would be embarrassing to get professional help when having a mental illness.

Personal stigma in terms of a wish for social distance (WSD) was negatively associated with help-seeking in a recent meta-analysis [12]. WSD seemed to lower the perceived need for professional help and, therefore, reduced the probability to be aware of one’s own illness; as a consequence, WSD minimized the likelihood to seek help [21]. Generally, WSD was more prominent in older than in younger persons with no differences between sexes [19]. Yet, stigma was a lower barrier to HSmental in studies with only female compared to only male participants [3]. In addition, perceived stigma has been negatively associated with help-seeking in adults [3] and with help-seeking intentions in adolescents [22]. In contrast to that, the intention to seek help was positively associated with actual HSmental [13, 23, 24]. Moreover, in a systematic review [11] but not in a longitudinal study [23], embarrassment about HSmental, i.e. anticipated stigma, was identified as a major barrier for HSmental in young people.

Most studies on HSmental were conducted only cross-sectionally [9, 12, 25] and frequently investigated only help-seeking intentions rather than actual help-seeking behaviour [6]. The longitudinal studies on HSmental, and stigma and attitudes have only a short follow-up of 6 months [8, 26, 27], small sample sizes [26], selected samples [28, 29], or, at a large follow-up of 11 years, focussed only on the impact of attitudes toward mental health help-seeking and beliefs about the effectiveness of treatment but not of personal and perceived stigma and low functioning [23]. Thus, longitudinal studies of sufficient sample size of the impact of stigma and attitudes, and their interaction on actual HSmental are clearly needed.

### Aims of the study

In order to address the lack of complex longitudinal studies on the impact of age, sex, various types of stigma, assumptions about own HSmental and functional deficit on actual HSmental behaviour within the following 3 years, we examined a complex structural equation model (SEM), which, based on the mainly cross-sectional findings described above, assumes the following effects on HSmental longitudinally:

- Perceived stigma is negatively associated with HSmental [3, 22].
- Personal stigma (WSD) is negatively associated with HSmental [13].
- Help-seeking intentions are positively associated with HSmental [13, 23, 24].
- Anticipated stigma (embarrassment about HSmental) is negatively associated with HSmental [11].
- Psychosocial functioning is negatively associated with HSmental [9].
- Past treatment experiences are positively associated with HSmental [9].
- Compared to male sex, female sex is more positively associated with HSmental [6, 30].
- Older age is positively associated with HSmental [6, 7, 9].

Considering not only direct but also indirect effects in our model, we aimed at detecting what predictors at baseline are linked to future help-seeking behaviour.

## Method

### Study design

**Fig. 1:**
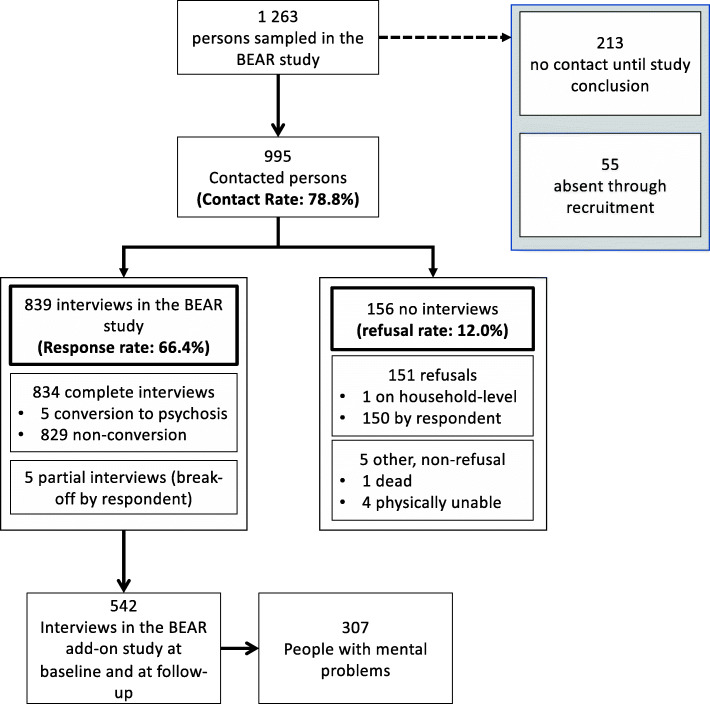
Recruitment process of the add-on study to the BEAR study according to the American Association for Public Opinion Research (AAPOR) Outcome Rate Calculator, version 3.1 [32]

Our study is based on the longitudinal data of an add-on study to the ‘Bern Epidemiological At-Risk’ (BEAR) study [31, 32]. At baseline, community participants of age 16 to 40 years were randomly drawn from the population register of the Canton Bern, Switzerland, first recruited for a telephone interview (response rate: 63.4%) and, at conclusion of the interview, for a questionnaire add-on study on stigma and mental health between June 2011 and June 2015 (*n* = 1519, response rate: 60.3%) [13]. At three-year follow-up, a preselected sub-sample of participants of the interview study were re-contacted between June 2015 and March 2018 (*n* = 1028, contact rate: 78.8%) [31] (Fig. 1). In the follow-up, 839 participants of the baseline interview study agreed to a second interview (response rate: 66.4%; see Fig. 1). Of these, 542 participants had participated in the add-on study at baseline and, thus, had available data on stigma and attitudes (Fig. 1). Since HSmental was our outcome variable of interest and the absence of HSmental in persons with and without mental health problems has to be evaluated differently – adequate behaviour in those without mental health problems but undesirable behaviour in those with mental health problems, we restricted our analyses to the 307 participants (56.6%) with mental disorders or relevant mental health problems past baseline, i.e., a positive response to any one screening question in the M.I.N.I. interview (Fig. 1).

For more information on recruitment see Additional file 1. For all studies, verbal informed consent was obtained and recorded from all subjects prior to both starting the telephone interview and posting the questionnaires. This procedure was chosen to avoid delays between first personal phone contact and posting of written consent that might have led to loosing contact with potential participants, in doing so decreasing recruitment rate and, thereby, generalizability. This procedure of obtaining consent verbally as well as all other procedures contributing to this work and involving human subjects were approved by the independent ethical committee of the University of Bern (No. 172/09) prior to starting the study. Furthermore, all procedures comply with the ethical standards of the relevant national and institutional committees on human experimentation and with the Helsinki Declaration of 1975, as revised in 2008.

### Assessments

Based on previous studies [33–35], life-time and current help-seeking was assessed by using a modified version of the WHO Pathway-to-Care questionnaire [33].

DSM-IV non-substance-related axis-I disorders were assessed at follow-up with the MINI-International Neuropsychiatric Interview’ (M.I.N.I) [36], which was previously used in telephone surveys and considered to be comparable with face-to-face interviews [37]. Thereby, the presence of any subthreshold mental health problems that signal a need of professional assessment and, consequently, help-seeking was assumed when a screening question was affirmed [38].

As a global rating of current functioning at baseline, the ‘Social and Occupational Functioning Assessment Scale’ (Sofas) [39] with scores ranging from 0 to 100, was used. Thereby, a score ≤ 70 was considered as an indication of deficient functioning [40, 41].

Personal stigma in terms of WSD was assessed at baseline with the adapted social distance scale developed by Link et al. [18]. In this, participants have to rate their willingness to socially interact in seven different situations with the person described in vignettes about patients with mental disorders (depression or schizophrenia) on a five-point Likert scale from 0 = ‘definitely willing’ to 4 = ‘definitely not willing’. Higher sum scores indicated stronger WSD.

Perceived stigma was assessed at baseline by presenting 10 items (Table 1) that describe public opinions about people with a mental illness (e.g. ‘Most people see it as an indicator of personal failure, when somebody is in a psychiatric clinic’). The participants had to rate these items on a five-point Likert scale from 1 = ‘nobody has this opinion’ to 5 = ‘everybody has this opinion’ according to the degree what they perceive as the public opinion.

**Table 1:** Sociodemographic and clinical characteristics of the sample (*n* = 307), and comparison of those who had sought help for mental health problems at follow-up and those who had not

	No help-seeking (*n* = 238; 77.5%)	Help-seeking (*n* = 69; 22.5%)	Total sample (*n* = 307)	Statistics U/χ^2^(df); Pearson’s r/ Cramer’s V
**Male sex, n (%)**	91 (38.2)	17 (24.6)	108 (35.2)	χ^2^_(1)_ = 4.337, *p* = 0.037, V = 0.119
**Age, median (mean ± SD)**	33 (31.25 ± 7.21)	31 (30.53 ± 7.72)	33 (31.09 ± 7.32)	U = 7936.500, *p* = 0.672, r = − 0.041
**Nationality, n (%) Swiss**	230 (96.6)	66 (95.7)	296 (96.4)	χ^2^_(1)_ = 0.151, *p* = 0.698, V = 0.022
**ISCED 2011**^**a**^**, n (%)**				χ^2^_(3)_ = 0.628 *p* = 0.731, V = 0.045
Primary education (ISCED 1)	2 (0.8)	1 (1.4)	3 (1.0)	
Secondary school (ISCED 2)	132 (55.5)	35 (50.7)	167 (54.4)	
High school (ISCED 3)	104 (43.7)	33 (47.8)	137 (44.6)	
**Employment, n (%)**				χ^2^_(3)_ = 11.658, *p* = 0.020, V = 0.195
Unemployed	5 (2.1)	3 (4.3)	8 (2.6)	
Protected employment	0 (0.0)	2 (2.9)	2 (0.7)	
Temporary work, self-employed	3 (1.3)	1 (1.4)	4 (1.3)	
Normal employment, in school	230 (96.6)	62 (89.9)	292 (95.1)	
**Marital status, n (%)**				χ^2^_(5)_ = 11.477, *p* = 0.043, V = 0.193
Unmarried	115 (48.3)	37 (53.6)	152 (49.5)	
Married or registered partnership	117 (49.2)	25 (36.2)	142 (46.3)	
Separated/divorced/widowed	2 (0.8)	3 (4.3)	5 (1.6)	
**Current non-psychotic axis-I disorder at baseline**^**b**^**, n (%)**	55 (23.1)	30 (43.5)	85 (27.7)	χ^2^_(1)_ = 11.085, *p* < 0.001, V = 0.190
**Current non-psychotic axis-I disorder at follow-up**^**b**^**, n (%)**	42 (17.6)	28 (40.6)	70 (22.8)	χ^2^_(1)_ = 15.982, *p* < 0.001, V = 0.228
**Current functional deficit at baseline**^**c**^	7 (2.9)	20 (29.0)	27 (8.8)	χ^2^_(1)_ = 45.234, *p* < 0.001, V = 0.384
**Current functional deficit at follow-up**^c^	10 (4.2)	23 (33.3)	33 (10.7)	χ^2^_(1)_ = 47.318, *p* < 0.001, V = 0.393
**Help-seeking at baseline, n (%)**	63 (26.5)	45 (65.2)	108 (35.2)	χ^2^_(1)_ = 35.218, *p* < 0.001, V = 0.339
**Intention to seek help**^**d**^**, n (%)**	199 (83.6)	61 (88.4)	260 (84.7)	χ^2^_(1)_ = 0.948, *p* = 0.330, V = 0.056

^a^ according to International Standard Classification of Education (ISCED) (UNESCO Institute for Statistics, 2012)^39^

^b^ according to Mini-International Neuropsychiatric Interview

^c^ defined as SOFAS score < 71

^d^ intention to seek help is dichotomized (0 = no or improbable intention to seek help, 1 = probable or definite intention to seek help)

In addition, anticipated stigma was measured by the question ‘How embarrassed would you be if your friends knew you were getting professional help for an emotional problem?’ with answer options ‘very embarrassed’ (=4), ‘somewhat embarrassed’ (=3), ‘not much embarrassed’ (=2), and ‘not at all embarrassed’ (=1).

Finally, help-seeking intentions were assessed by the question ‘If you had a serious emotional problem, would you seek professional help?’ with answer options ‘definitely’ (=4), ‘probably’ (=3), ‘probably not’ (=2), and ‘definitely not all embarrassed’ (=1). Finally, actual help-seeking behavior was binary assessed by the questions ‘Have you ever sought help for mental health problems?’ (at baseline) and ‘Have you sought help for mental health problems since the first interview?’ (at follow-up).

### Statistical analyses

Categorical data were compared by χ^2^-tests, non-normally distributed ratio and ordinal data by Mann-Whitney U tests. Principal component analyses (PCA) with Varimax rotation were conducted on the 10 items on perceived stigma. Thereby, we used pairwise complete observations to deal with missing values. Next, the Kaiser-Meyer Olkin (KMO) measure was used to check the sampling adequacy for the analyses.

Secondly, we conducted a theoretically grounded SEM, which was based on the mainly cross-sectional findings described in the aims section. Within the SEM, we had 2% missing items. Based on the results of the PCA and previous studies, we formed one latent variable for ‘perceived stigma’. The variables ‘WSD’ [19], ‘help-seeking intention’ [13, 23, 24], ‘anticipated stigma [11], ‘age’ [7], ‘sex’ [30], ‘functional deficit’ [9], ‘help-seeking at baseline’ [9, 10, 13] were modelled as observed variables. The pathways from stigma via own help-seeking assumptions (i.e., help-seeking intentions) to help-seeking behaviour including all likely associations between latent and manifest variables were modelled in a SEM. To examine the bivariate relationships between the variables, we calculated Spearman’s correlation coefficients. To test also for indirect effects, we built mediation pathways within the model by labelling potential parameters in the regression as parameters. The statistical analyses were conducted in SPSS 25.0 and in the R language for statistical computing using the packages “lavaan” [42] and “psych” [43], respectively. Throughout, we considered a level of significance of α < .05.

## Results

### Power analysis and sample characteristics

The calculation of the model was performed with *N* = 307 participants with mental health problems or disorders. A power analysis of the final model, which contained 12 variables and df = 27 degrees of freedom, resulted in a power of 0.901 with an RMSEA = 0.06 and an α = 0.05. Of the 307 participants, 238 (77.5%) participants had not and 69 (22.5%) participants had sought help past baseline (Table 1). The average age was around 33 years with no difference between help-seekers and non-help-seekers. Revealing a small effect of sex, more female than male participants had sought help (Table 1). The majority of the participants were Swiss, frequently unmarried, and had a secondary school education (Table 1). There was a small effect towards help-seekers being more frequently unemployed or working in protected employment (Table 1). In addition, help-seekers had more frequently functional deficits at baseline or follow-up, and had more frequently reported HSmental already at baseline; all these differences revealed moderate effect size (Table 1). Help-seekers also had more often any current non-psychotic axis-I disorder at baseline or follow-up; yet, differences only revealed small-to-moderate effects (Table 1). Help-seekers, however, had not more frequently reported help-seeking intentions at baseline; this difference showed a moderate effect size (Table 1).

### Factors of perceived stigma

In the PCA of the 10 perceived stigma items, the KMO measure indicated excellent or “meritorious” [44] sampling adequacy for the analyses (KMO = .85), and all KMO values for individual items were > .80 in the PCA, and thus above the acceptable limit of .5 [45]. Bartlett’s test of sphericity (χ^2^_(45)_ = 857.186, *p* < 0.001) indicated that correlations between items were sufficiently large for PCA [45]. Two independent factors (‘perceived stigma, ‘no perceived stigma’) had an eigenvalue over Kaiser’s criterion of 1 and explained 50% of the variance (Table 2).

**Table 2:** Results of the principal component analysis (PCA) of the 10 items asking about perceived stigma (*N* = 307). Only factor loadings > 0.40 are displayed

Items	Factor 1: no perceived stigma	Factor 2: perceived stigma
Most people have no problems to be the friend of a former psychiatric patient.	0.40	
Most people believe that somebody who was in a psychiatric clinic is just as intelligent as the average population.	0.62	
Most people have the opinion that one can trust a former psychiatric patient just like other people.	0.61	
Most people agree that a psychiatrically treated person, who is totally recovered, can be a teacher for little children.	0.44	
Most people see it as an indicator of personal failure, when somebody is in a psychiatric clinic.		0.68
Most people do not allow former psychiatric patients to look after their children, even if they are doing well for some time.		0.63
Most people think less of somebody who was in a psychiatric clinic.		0.78
Most employees hire a former psychiatric patient when he or she is qualified for the job.	0.62	
Most people treat former psychiatric patients just like other people.	0.63	
Most people do not take the opinion of somebody who was in a psychiatric clinic seriously.		0.55
**Eigenvalue**	2.00	1.88

### Association between stigma, assumptions about own help-seeking, and healthcare utilization

**Fig. 2:**
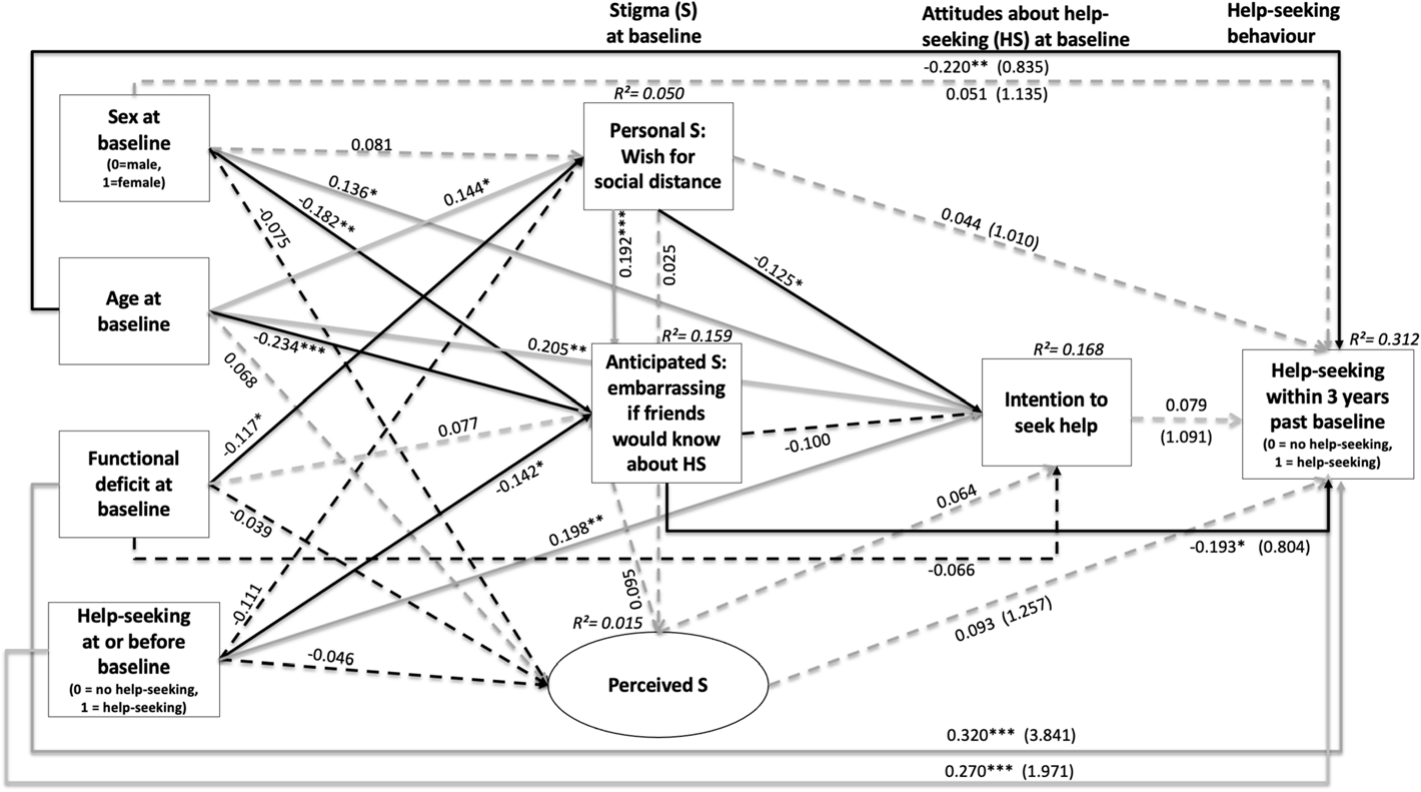
Final model of associations between stigma, assumptions about help-seeking and healthcare utilization with standardized path coefficients (*N* = 307)

The fit indices of our longitudinal model (Fig. 2), CFI (guide value ≥0.95), RMSEA (guide value ≤0.06), SRMR (guide value ≤0.08) and 90% confidence intervals not containing 0.08) suggested good fit of our model to data [46, 47]. As previously recommended [48, 49], no post-hoc modifications were conducted but, for better overview, a model with only significant paths is displayed in the Additional file 2.

In our model (Fig. 2), HSmental at follow-up was mostly related to functional deficit and HSmental at baseline but also, though to a lesser degree, to younger age and lower anticipated stigma at baseline. Furthermore, age exerted several other influences on other baseline variables: Older age was related to both stronger WSD and more help-seeking intentions, while younger age was related to more anticipated stigma. Sex did not influence HSmental at follow-up, but anticipated stigma was higher in males and help-seeking intentions were higher in females. Yet, unexpectantly, both baseline help-seeking intentions and baseline personal stigma in terms of WSD were not significantly related to HSmental at follow-up, yet, WSD decreased help-seeking intentions and increased anticipated stigma. In order to examine an indirect effect of personal stigma on HSmental at follow-up via anticipated stigma, we tested the pathway “personal stigma – anticipated stigma – HSmental at follow-up”; however, this also remained insignificant (*p* = 0.438). Perceived stigma at baseline was completely unrelated to any other variable in the model (Fig. 2).

Model fit indices: χ^2^_(27)_ = 32.174 with *p* = 0.226, CFI = 0.985; SRMR = 0.047; RMSEA = 0.025 (90%CIs = 0.000, 0.054).

*p ≤ 0.05; **p ≤ 0.01; ***p ≤ 0.001; explained variance (R^2^) for each endogenous variable in italics. In brackets, Odds Ratios for the endogenous variable “help-seeking within 3 years past baseline” are provided. Manifest variables are represented in rectangles, latent ones in ovals. Solid lines indicate significant paths, dashed lines indicate non-significant paths; in doing so, grey indicates positive, black negative correlations.

The bivariate correlations of the variables of the model are given in Table 3. Contrary to the SEM, they indicated small to moderate correlations between HSmental at follow-up with help-seeking intentions and with female sex but not age. In line with the model, functional deficits and earlier HSmental as well as anticipated stigma, but not personal or perceived stigma were significantly related to subsequent HSmental. Personal and perceived stigma were also uncorrelated to HSmental at baseline (Table 3).

**Table 3:** Spearman-correlations of variables used in the model (*n* = 307)

	1.	2.	3.	4.	5.	6.	7.	8.
1. Sex	1.0							
2. Age at baseline	−.04	1.0						
3. Functional deficit	.04	.00	1.0					
4. Help-seeking at baseline	.14*	.22***	.33***	1.0				
5. Personal stigma (WSD)	.04	.11*	−.13*	.09	1.0			
6. Perceived stigma	−.03	.05	−.07	.01	.07	1.0		
7. Anticipated stigma	−.16**	−.22***	−.02	.20***	.16**	.02	1.0	
8. Intention to seek help	.16**	.24***	.02	.25***	−.09	.06	−.22***	1.0
9. Help-seeking after 3 years	.12*	−.04	.38***	.34***	−.08	.02	−.14*	.12*

* *p* < .05; ** *p* < .01; *** *p* < .001

## Discussion

Our unique longitudinal study examined the complex associations of various types of stigma (personal, perceived and anticipated), help-seeking intentions, functional deficit and health care utilization in a community sample of persons with mental health problems using structural equation modelling. Contrary to the longitudinal studies with 3- and 6-month follow-ups [8, 26, 27], our follow-up period of 3 years, was sufficiently long to allow for the new emergence of mental health problems and related help-seeking. While in comparison to a study with 11-year follow up of persons with and without mental health problems [23], our study was still short enough to rule out significant recall bias and significant change in attitudes and did not confuse the interpretation of non-help-seeking by mixing persons with and without mental health problems. Furthermore, our sample was randomly selected from the general population of, at baseline, 16–40-year-olds, i.e., in an age range in that many mental disorders develop first [50] and sufficiently large to ensure good power. Thus, our results are likely more generalizable to Middle-European samples in an age range of highest risk to develop a first episode of mental illness than the ones reported from the much smaller, rather old convenience sample (*N* = 188; mean age: 50 years) of the 3- and 6-month follow-up studies that was also preselected for symptoms of depression only [8, 26].

Our longitudinal analysis revealed expected associations of functional deficits and of earlier help-seeking for mental health problems with subsequent help-seeking that were similar to those cross-sectionally reported earlier from a larger baseline sample of the BEAR study [9]. Furthermore, it supported the negative effect of anticipated stigma on help-seeking for mental health problems [11]. With regard to age, the reported association of older age with help-seeking [6, 7, 9] was not supported by our study, in which the contrary effect, an association with younger age, was found. Other direct effects on help-seeking reported from cross-sectional studies could not be replicated longitudinally, such as the help-seeking reducing effect of perceived and personal stigma [3, 13, 22], which also did not show on the level of bivariate correlations, or the help-seeking increasing effect of earlier help-seeking intentions [13, 23, 24], and of female sex [6, 8].

In line with previous results [9], our results indicated that functional deficits had the strongest effect on help-seeking for mental health problems at follow-up in both bivariate correlations and the model (Fig. 2). This indicates that persons with mental health problems are more likely to seek help, when they already experience functional impairments in some areas of life. These, however, only develop over the course of the disorder and/or when problems of multiple domains of mental disorder have already developed [9]. This important role of functional impairments in help-seeking for mental health problems is unfortunate in light of the fact that early help-seeking for mental health problems, in particular, is a prerequisite not only for preventing mental disorder but also for preventing impairments in functioning and quality of life [4].

The second most important predictor of help-seeking for mental health problems at follow-up was help-seeking for mental health problems at baseline, which is in line with other studies [9, 19]. This might reflect an effect of familiarity and previous positive experience with mental health services that counteracts negative beliefs towards mental health services and professionals, which were found to be the most cited barriers to help-seeking in a recent review focussing on adolescents [10]. Thus, future longitudinal studies in even larger samples should also focus on the interplay between these variables and their predictors, including for example the type of mental health problems that help was sought for. This might be important in light of reports that help-seeking is predominately reported because of depressiveness, anxiety and interpersonal problems [51], possibly because these symptoms were reported to impact most strongly and persistent on self-perceived health status and quality of life [52], which might also affect help-seeking.

Contrary to some cross-sectional studies [3, 22], but in line with a recent meta-analysis on the impact of various stigma types on help-seeking [12], perceived stigma had no significant effect on help-seeking or assumptions about own help-seeking. In the meta-analysis [12], only personal attitudes towards mental illness or help-seeking were significantly associated with active help-seeking, in particular own negative attitudes towards help-seeking (including anticipated stigma in terms of embarrassment about help-seeking) and personal stigma (including WSD). In our model, these effects were not independent of each other; rather, contrary to our hypotheses, personal stigma had no direct effect on help-seeking for mental health problems at follow-up, but increased anticipated stigma at baseline. Furthermore, anticipated stigma significantly decreased help-seeking for mental health problems at follow-up. This finding is contrary to that of another longitudinal study [23], in which anticipated stigma had no influence on future help-seeking for mental health problems. However, this study was an 11-year follow-up and, thus, this particular assumption about help-seeking might have changed over the 11 years, because anticipated stigma about help-seeking was more prominent in younger people in our model.

Despite the weak but significant correlation that is in line with earlier reports on a positive association between help-seeking intentions and help-seeking [13, 23, 24], help-seeking intentions were not related to help-seeking for mental health problems at follow-up in our model. This might be due to factors that might influence the association between help-seeking intentions and help-seeking differently over time, such as the perceived need for help-seeking [24], perceived accessibility, spatial and temporal distance from mental health services, treatment efficacy beliefs, and anticipated self-stigma [8] that should be studied in more detail in future studies. The impact of the perceived need for help-seeking, however, might be partially reflected in the independent role of functional deficit, the strongest predictor of help-seeking for mental health problems at follow-up, whereat the association between perceived need for help-seeking and functioning might be further moderated by aspects of autonomy and underestimation of symptoms [53]. Yet, the gap between intention and behaviour is well known [54] and clearly observable in our study, in which 84.7% of the persons with mental health problems stated an intention to seek professional help for a serious mental problem, but only 19.9% actually sought help. Thus, future research should carefully differentiate between help-seeking intention and behaviour, and focus more on the latter.

In line with previous findings [6], women had higher intention to seek help; yet, a sex effect on help-seeking for mental health problems could not be detected, albeit frequent report of more help-seeking for mental health problems in women [6, 55] and the significant positive correlation between female sex and help-seeking for mental health problems. This indicates that the frequently reported sex effect on help-seeking for mental health problems may rather be mediated by other factors such as anticipated stigma about help-seeking, which was less severe in females.

### Strengths and limitations

Besides the strengths of our well-powered study of being one of the very few longitudinal community studies and of observing multiple factors and their interrelations in one model of help-seeking in persons of the community with mental health problems, some limitations have to be discussed. First, our sample was restricted to German-speaking persons because of the language of the questionnaires and to persons between 16 to 40 years at baseline because of the study focus of the BEAR study on psychosis-risk symptoms; additionally, it was of mainly Middle-European background. Due to existing cultural differences regarding help-seeking [56] and stigma [3], our study may thus only generalize to the Western, Middle-European culture. Another limitation, which interview- or questionnaire-based studies usually have in common, is the probability of systematic response bias due to social desirability. Furthermore, as already discussed above, our model did not include all possible moderators of help-seeking for mental health problems itself as well as of some of the significant factors in the model.

## Conclusion

With only 22.5% of persons with mental health problems seeking any help for these, our study confirmed a prominent treatment gap. Functional deficits, which may introduce a perceived need for help, had the strongest impact on help-seeking for mental health problems longitudinally. In doing so, younger men showed more anticipated stigma towards help-seeking at follow-up, which decreased help-seeking for mental health problems at follow-up. Surprisingly, personal and perceived stigma had no direct effect on help-seeking for mental health problems, nor had help-seeking intentions or sex. At a non-help-seeking rate of more than 75%, our study raises the questions, whether the health care system is offering enough low-threshold help-seeking opportunities for persons with emerging mental health problems, and how to design mental health care systems that are not associated with anticipated stigma, i.e., that identify features conveying anticipated stigma.

## Supplementary Information

**Additional file 1.**

**Additional file 2.**

## Data Availability

The datasets used and/or analysed during the current study are available from the corresponding author on reasonable request.

## Abbreviations

AAPOR: American Association for Public Opinion Research
BEAR: Bern Epidemiological At-Risk
HSmental: Help-seeking for mental health problems
WSD: Wish for social distance
SEM: Structural equation model
