# Supplementary material for: Predictors of help-seeking behaviour in people with mental health problems: a 3-year prospective community study
Source: BMC Psychiatry. 2021 Sep 3;21:432. doi: 10.1186/s12888-021-03435-4 (PMC8414662; doi:10.1186/s12888-021-03435-4)
Supplement: Supplementary file 1 — Additional file 1. [file 12888_2021_3435_MOESM1_ESM.pdf]

Additional information file

for

Predictors of help-seeking behaviour in people with mental health problems: a 3-year prospective community study

Carolin M. Doll[1,2], Chantal Michel[3], Marlene Rosen[2], Naweed Osman[1], Benno G. Schimmelmann[3,4], Frauke Schultze-Lutter[1,3,5]

1 Department of Psychiatry and Psychotherapy, Medical Faculty, Heinrich-Heine-University, Düsseldorf, Germany. Electronic address: carolin.doll@uk-koeln.de

2 Department of Psychiatry and Psychotherapy, Faculty of Medicine and University Hospital Cologne, University of Cologne, Cologne, Germany.

3 University Hospital of Child and Adolescent Psychiatry and Psychotherapy, University of Bern, Bern, Switzerland.

4 University Hospital of Child and Adolescent Psychiatry, University Hospital Hamburg-Eppendorf, Hamburg, Germany.

5 Department of Psychology and Mental Health, Faculty of Psychology, Airlangga University, Surabaya, Indonesia

Correspondence author:

Carolin Doll, M.Sc.

Department of Psychiatry and Psychotherapy

Faculty of Heinrich- Heine University and LVR clinic Düsseldorf

Bergische Landstr. 2, 40629 Düsseldorf

Email: carolin.doll@uk-koeln.de, phone: +49221 478 7225

ORCID: https://orcid.org/0000-0002-4267-1668

eText 1 Details on study design

To increase response rates, contact was initially established using a one-page information letter at both baseline and follow-up. First telephone contact was attempted within two weeks of sending the letter. After detailed explanation of goals and proceedings of the study, participation in the telephone interview was considered as giving informed consent.

At baseline, inclusion criteria were being of eligible age (16-40 years) and being a main resident of Canton Bern (i.e. having a valid address and not being abroad during the assessment period). At follow-up, inclusion criteria were participation in baseline interview and consent to be re-contacted. In addition. an available telephone number was required for eligibility at both baseline and follow-up. We called participants up to 100 times over several months at various times and days, including Saturdays. Potential participants that were not reached within this time were considered as unknown eligible. Moreover, interviews at both baseline and follow-up were aborted prematurely when respondents had (i) a lifetime diagnosis of psychosis (1) or, at baseline, (ii) insufficient language skills in German, French, or English.

The semi-structured interviews lasted 43 minutes on average (SD: 20 minutes; range: 20–225 minutes) at baseline, and 52 minutes on average (SD: 26 minutes; range: 24-248 minutes) at follow-up.

The BEAR study was carried out in accordance with the latest version of the Declaration of Helsinki. Further details on recruitment and sample at baseline and follow-up are provided in Schultze-Lutter and colleagues [2,3].

Details on recruitment of sample and representativeness

Baseline assessment

At baseline, from the 4,471 eligible participants, 2,857 interviews were conducted [2]. However, 125 (4.4%) of the 2,857 interviews were aborted prematurely by the interviewer for insufficient language skills; 41 (1.4%) were aborted for a lifetime diagnosis of psychosis, in which 19 were not diagnosed/treated; [1] and 8 (0.3%) were terminated prematurely by the participants themselves. Lack of time or interest was the main reason given by the 1,350 (29.5%) refusers.

The contact rate was 94.8%, and the response rate, 63.4% with 2,683 completed interviews. The eligible sample was negligibly older than the 16- to 40-year-old general population of Bern, mainly because a non-significant higher number of available telephone numbers (landlines) was found for 36- to 40-year-olds. Similar to the observation for the eligibility sample, the 2,683 interviewees differed negligibly, i.e., at less than small effect size, from the 16- to 40-year-old general population of Bern in age distribution, but not in sex, nationality, and marital status. Consequently, as no response bias was detectable beyond the extremely small age-related inclusion bias, the interviewees were regarded as representative of their age group [2].

Of the 2,683 interviewees, 2,539 were sufficiently fluent in German to be eligible for the add-on questionnaire study of mental health literacy and stigma. Of these, 324 refused to participate. Of the 2,215 interviewees who agreed to the add-on study, 689 did not returned the questionnaire after a maximum of three reminder calls, while 1,526 returned the questionnaire. Thus, according to the definitions of the American Association for Public Opinion Research [4], the contact rate of the add-on study was 72.9%, the cooperation rate 82.5%, the refusal rate 12.8%, and the response rate 60.1%.

Follow-up assessment

Originally, the follow-up recruitment period was planned to span over 36 months. However, due to the PI (FSL) leaving Switzerland, funding stopped slightly earlier, and the study was concluded two months earlier. Thus, during the abbreviated 34 months of recruitment (June 2015-March 2018), the original recruitment aims of n=500 persons who had reported at least one lifetime clinical high risk (CHR) symptom (RISK) at baseline and n=500 matched persons who had not reported any CHR symptom (CONTROL) were not fully reached, and the contact rate was only 78.8% (eFigure 3). Furthermore, until conclusion of the study, contact with the target person could only be re-established in 995 persons, resulting in a cooperation rate of 84.7% (eFigure 3). Of these 995 persons, 151 refused to participate again in the study (eFigure 3). The main reasons for refusal given by the n=86 (57.0%) refusers who agreed to participate in a non-responder interview were similar to those given by refusers of the baseline [2]. In descending order and with multiple answers possible, reasons for refusal at follow-up were: 46 (53.5%) lack of time, 37 (43.0%) lack of interest, 11 (12.8%) interview too long, 10 (11.6%) too intimate and/or private questions, 6 (7.0%) no personal gain, 3 (3.5%) irrelevant topic.

Of the 834 interviewees with a full interview [3], 434 had been sampled as RISK subjects (52.0%) and 400 as CONTROL subjects (48.0%). Thus, slightly more sampled RISK than sampled CONTROL had participated until early study termination ($\chi^2_{(1)}$=6.832, p=0.009, Cramer's V=0.074). However, interviewed RISK and CONTROL did not differ in baseline age (RISK: 30.4±7.7 yrs., Mdn=32.2 yrs.; CONTROL: 30.2±7.7 yrs., Mdn=32.4 yrs.; U=85384.5, p= 0.684, Rosenthal's r=0.014), sex (RISK: 46.5% male, CONTROL: 47.3% male; $\chi^2_{(1)}$=0.042, p=0.838, Cramer's V=0.007), baseline nationality (RISK: 96.3% Swiss, CONTROL: 96.3% Swiss; $\chi^2_{(1)}$=0.002, p=0.961, Cramer's V=0.002), baseline highest educational level (RISK: 86.3% ISCED 5 or higher, CONTROL: 86.1% ISCED 5 or higher; $\chi^2_{(1)}$=4.549, p=0.715, Cramer's V=0.074), and baseline partnership (RISK: 56.9% single, CONTROL: 56.8% single; $\chi^2_{(1)}$=0.002, p=0.962, Cramer's V=0.002) [3]. Thus, despite the pre-term conclusion of the study and the negligible bias towards recruitment of RISK, matching was sufficient and both samples were well comparable.

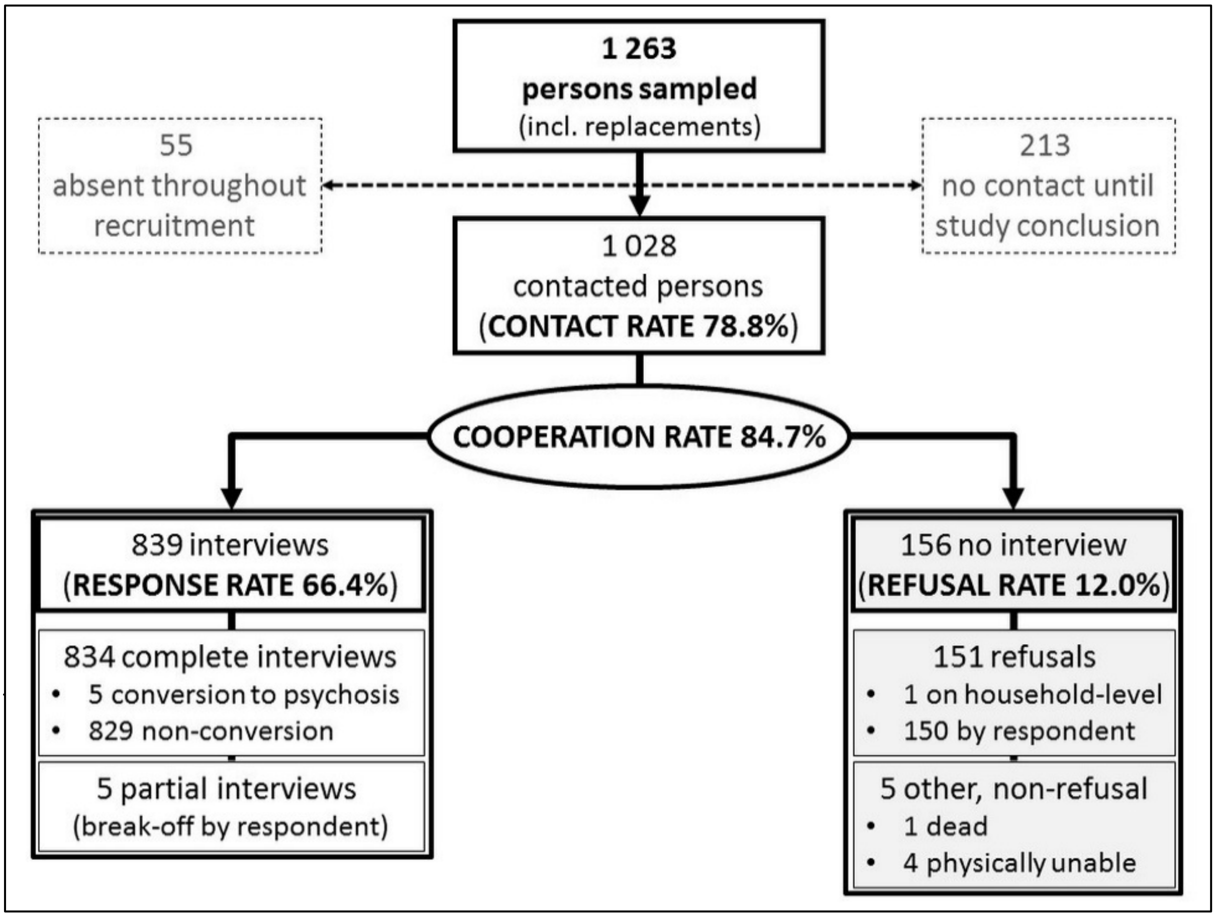

eFig. 3. Survey outcome rates of the first follow-up of the BEAR study according to the definitions of the American Association for Public Opinion Research, AAPOR [4].

Of the 834 participants with a complete follow-up interview, 542 (65.0%) had participated in the add-on study at baseline and returned the questionnaire. They formed the sample of the analyses in the present study.

References

1. Michel C, Schimmelmann BG, Schultze-Lutter F. Demographic and clinical characteristics of diagnosed and non-diagnosed psychotic disorders in the community. Early intervention in psychiatry. 2018;12(1): 87-90.
2. Schultze-Lutter F, Michel C, Ruhrmann S, Schimmelmann BG. Prevalence and clinical relevance of interview-assessed psychosis-risk symptoms in the young adult community. Psychol Med. 2018;48(7):1167-78.
3. Schultze-Lutter F, Schimmelmann BG, Michel C. Clinical high-risk of and conversion to psychosis in the community: A three-year follow-up of a cohort study. Schizophrenia Research. 2021;228:616-8.
4. American Association for Public Opinions research. Standard Definitions. Final Dispositions of Case Codes and Outcome Rates for Surveys. 9th edition: AAPOR; 2016.
