# Supplementary material for: Predictors of help-seeking behaviour in people with mental health problems: a 3-year prospective community study
Source: BMC Psychiatry. 2021 Sep 3;21:432. doi: 10.1186/s12888-021-03435-4 (PMC8414662; doi:10.1186/s12888-021-03435-4)
Supplement: Supplementary file 2 — Additional file 2. [file 12888_2021_3435_MOESM2_ESM.pdf]

Additional information file

for

Predictors of help-seeking behaviour in people with mental health problems: a 3-year prospective community study

Carolin M. Doll[1,2], Chantal Michel[3], Marlene Rosen[2], Naweed Osmann[1], Benno G. Schimmelmann[3,4], Frauke Schultze-Lutter[1,3,5]

1 Department of Psychiatry and Psychotherapy, Medical Faculty, Heinrich-Heine-University, Düsseldorf, Germany. Electronic address: carolin.doll@uk-koeln.de

2 Department of Psychiatry and Psychotherapy, Faculty of Medicine and University Hospital Cologne, University of Cologne, Cologne, Germany.

3 University Hospital of Child and Adolescent Psychiatry and Psychotherapy, University of Bern, Bern, Switzerland.

4 University Hospital of Child and Adolescent Psychiatry, University Hospital Hamburg-Eppendorf, Hamburg, Germany.

5 Department of Psychology and Mental Health, Faculty of Psychology, Airlangga University, Surabaya, Indonesia

Correspondence author:

Carolin Doll, M.Sc.

Department of Psychiatry and Psychotherapy

Faculty of Heinrich- Heine University and LVR clinic Düsseldorf

Bergische Landstr. 2, 40629 Düsseldorf

Email: carolin.doll@uk-koeln.de, phone: +49221 478 7225

ORCID: https://orcid.org/0000-0002-4267-1668

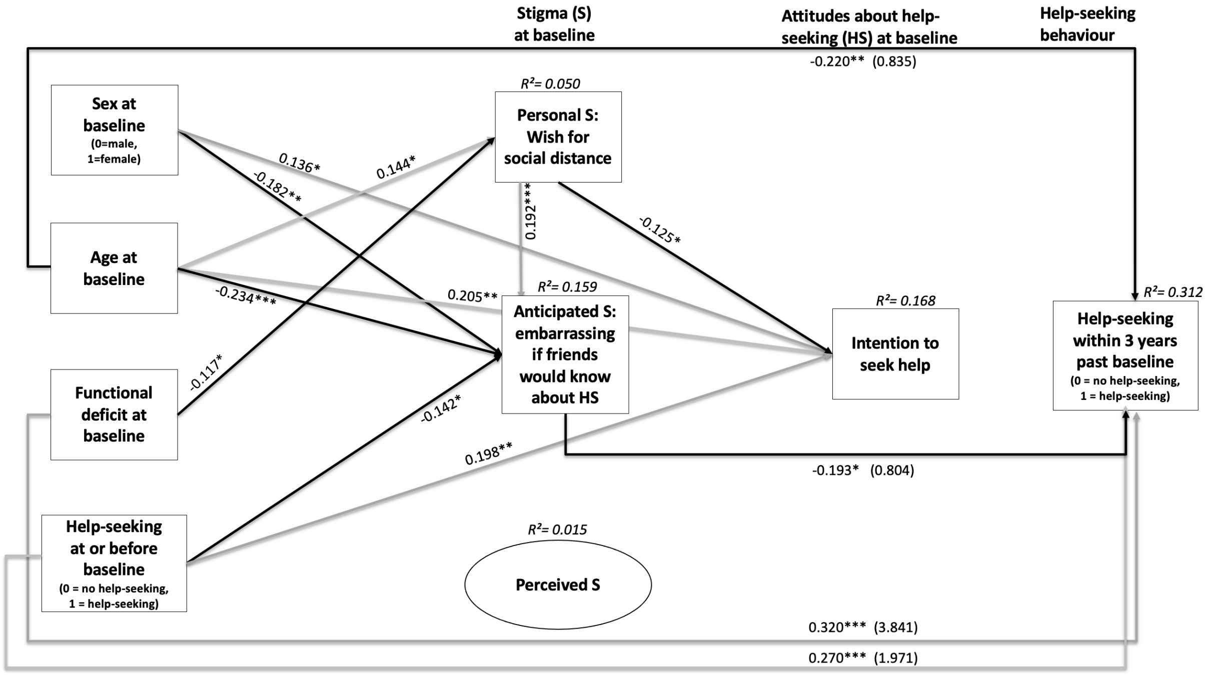

eFig. 2.

Final model of only significant associations between stigma, assumptions about help-seeking and healthcare utilization with standardized path coefficients (N=307).

Model fit indices: $\chi^2_{(27)}$=32.174 with p=0.226, CFI=0.985; SRMR=0.047; RMSEA=0.025 (90%CIs=0.000, 0.054). *p≤0.05; **p≤0.01; ***p≤0.001; explained variance (R^2) for each endogenous variable in italics. In brackets, Odds Ratios for the endogenous variable "help-seeking within 3 years past baseline" are provided. Manifest variables are represented in rectangles, latent ones in ovals. Solid lines indicate significant paths, dashed lines indicate non-significant paths; in doing so, grey indicates positive, black negative correlations.
